# Safety of Recombinant VSV–Ebola Virus Vaccine Vector in Pigs

**DOI:** 10.3201/eid2104.142012

**Published:** 2015-04

**Authors:** Emmie de Wit, Andrea Marzi, Trenton Bushmaker, Doug Brining, Dana Scott, Juergen A. Richt, Thomas W. Geisbert, Heinz Feldmann

**Affiliations:** National Institute of Allergy and Infectious Diseases, Hamilton, Montana, USA (E. de Wit, A. Marzi, T. Bushmaker, D. Brining, D. Scott, H. Feldmann);; Kansas State University College of Veterinary Medicine, Manhattan, Kansas, USA (J.A. Richt);; University of Texas Medical Branch, Galveston, Texas, USA (T.W. Geisbert);; University of Manitoba, Winnipeg, Manitoba, Canada (H. Feldmann)

**Keywords:** Ebola virus, viruses, vesicular stomatitis viruses, VSV, recombinant VSV–Ebola virus vaccine vector, VSV vaccine, zoonoses, pigs, safety, vaccines

## Abstract

The ongoing Ebola outbreak in West Africa has resulted in fast-track development of vaccine candidates. We tested a vesicular stomatitis virus vector expressing Ebola virus glycoprotein for safety in pigs. Inoculation did not cause disease and vaccine virus shedding was minimal, which indicated that the vaccine virus does not pose a risk of dissemination in pigs.

The current Ebola virus (EBOV) outbreak in West Africa has shown the need for an effective vaccine against this virus. As a result, clinical trials to test several vaccine candidates have been expedited (*1*) in hopes of contributing to containment of the outbreak. One of these vaccine candidates is based on a recombinant vesiculovirus vector, species vesicular stomatitis Indiana virus (here designated and more commonly known as VSV) expressing the EBOV strain Mayinga glycoprotein (here designated rVSV∆G/EBOVGP; formerly designated VSV∆G/ZEBOVGP) (*2*–*4*). This vaccine was highly efficacious in preexposure and postexposure studies in nonhuman primates after a single injection (*5*). In addition, the vaccine has been shown to be safe in simian HIV–infected rhesus macaques (*6*) and was not neurovirulent after intrathalamic inoculation into macaques (*7*).

However, because VSV is a World Organisation for Animal Health–listed pathogen (*8*), concerns might arise with regard to spillover of the vaccine vector to livestock when this vaccine is used on a larger scale in humans. To evaluate the safety of rVSV∆G/EBOVGP in a relevant livestock species, we inoculated pigs with this vaccine and compared clinical signs and virus replication with those of a recombinant wild-type VSV vector (rVSVwt) described previously (*3*). 

## The Study

All animal experiments were approved by the Institutional Animal Care and Use Committee of the Rocky Mountain Laboratories and performed following the guidelines of the Association for Assessment and Accreditation of Laboratory Animal Care, International. Experiments were performed by certified staff in an Association for Assessment and Accreditation of Laboratory Animal Care AAALAC–approved facility, following the guidelines and basic principles in the US Public Health Service Policy on Humane Care and Use of Laboratory Animals and the Guide for Care and Use of Laboratory Animals.

Four-week old pigs (Yorkshire cross) were obtained from the Washington State University College of Veterinary Medicine (Pullman, WA, USA). One group of 5 pigs and 1 group of 6 pigs were inoculated with rVSVwt and rVSV∆G/EBOVGP, respectively, as controls; 2 animals were mock inoculated with culture medium (Dulbecco modified Eagle medium). Animals were inoculated with 10^6^ PFUs of either virus in a 100-μL volume, or an equal volume of Dulbecco modified Eagle medium by intradermal injection in the apex of the snout (*9*).

At regular intervals after inoculation, clinical examinations were performed to determine the health status of the animals and to collect nasal, throat, and rectal swab samples for virologic analysis; blood was collected to determine the humoral immune response. Three animals inoculated with rVSVwt and rVSV∆G/EBOVGP were euthanized at 3 days postinoculation (dpi) as per protocol; the remaining animals were euthanized at 21 dpi.

Inoculation of pigs with rVSVwt and rVSV∆G/EBOVGP did not result in obvious signs of disease (Table), changes in body temperature, or a decrease in weight gain compared with mock-inoculated controls. A nose lesion developed at 4 dpi at the injection site in 1 animal inoculated with rVSVwt, but this lesion healed by 9 dpi. Swab specimens collected from the lesion site on 5, 6, 7, 8, and 10 dpi were negative by virus titration. Nose, throat, and rectal swab specimens were collected at 1, 3, 6, 10, 14, and 21 dpi; a nose swab specimen collected at 3 dpi from a pig inoculated with rVSV∆G/EBOVGP was the only specimen in which virus could be detected (virus titer 10^0.83^ 50% tissue culture infectious dose [TCID_50_]/mL) (Table).

**Table:** Findings for pigs inoculated with rVSVwt and rVSV∆G/EBOVGP*

Inoculum	Clinical signs	VSV lesions	Virus shedding from			Viremia	Virus replication in tissues	Seroconversion at 21 dpi	
			Nose	Throat	Rectum			VSV-G	EBOVGP
Mock (control)	ND	ND	ND	ND	ND	ND	ND	ND	ND
rVSVwt	ND	1/5†	ND	ND	ND	ND	2/5‡	2/2	ND
rVSV∆G/EBOVGP	ND	ND	ND	1/6§	ND	ND	2/6¶	ND	3/3

*rVSVwt, recombinant wild-type vesicular stomatitis virus; rVSV∆G/EBOVGP, recombinant VSV expressing Ebola virus strain Mayinga glycoprotein; VSV-G, VSV glycoprotein; dpi, day postinoculation; ND, not detected. †Lesion at inoculation site. ‡Snout positive in virus titration in 2 of 3 animals at 3 dpi. §One swab collected from 1 animal positive at 3 dpi. ¶Snout positive in 1 of 3 animals at 3 dpi; inguinal lymph node positive in 1 of 3 animals at 3 dpi.

Three animals in each group were euthanized at 3 dpi. Tissue samples from lip, tongue, snout, footpad, coronary band, interdigital skin, tonsil, oronasopharynx, inguinal lymph node, axillary lymph node, cervical lymph node, mesenteric lymph node, bronchial lymph node, nasal mucosa, trachea, bronchus, lungs, heart, liver, spleen, kidney, adrenal gland, pancreas, jejunum, colon, urinary bladder, cervical spinal cord, frontal brain, cerebellum, and brain stem of these animals were collected for virus titration and histologic analysis.

In 2 of 3 animals inoculated with rVSVwt, virus was detected in the snout (virus titers 10^2.3^ and 10^2.6^ TCID_50_/g), but virus was not detected in any of the other tissues collected at 3 dpi. In 1 of the pigs inoculated with rVSV∆G/EBOVGP, virus was detected in the snout (virus titer 10^2.5^ TCID_50_/g) and in another pig in an inguinal lymph node (virus titer 10^5.1^ TCID_50_/g). This high titer in the inguinal lymph node might have been the result of a change in cell tropism caused by use of EBOVGP rather than VSV glycoprotein (VSV-G). Histologic analysis did not identify lesions consistent with VSV infection in the rVSVwt– or rVSV∆G/EBOVGP–inoculated animals. At the end of the experiment at 21 dpi, the same tissues were collected for virologic and histologic analysis; virus could no longer be detected in any of the tissues derived from the inoculated animals, and no histologic lesions were present.

Serum samples collected at 21 dpi were analyzed for IgG against VSV-G or EBOVGP by using an IgG ELISA and secreted forms of these glycoproteins as antigens (*10*,*11*), respectively. In the VSV-G ELISA, pigs inoculated with rVSVwt showed an antibody response to VSV-G at 21 dpi (titers 1:400 and 1:1600); animals inoculated with rVSV∆G/EBOVGP did not show seroconversion to VSV-G. In an ELISA specific for the EBOVGP, pigs inoculated with rVSV∆G/EBOVGP showed a robust antibody response (titers 1:800, 1:1,600, and 1:3,200 by 21 dpi), but animals inoculated with rVSVwt did not show seroconversion to EBOVGP.

## Conclusions

Our data indicate that, although rVSV∆G/EBOVGP can replicate in pigs, this vaccine virus does not result in overt clinical disease, and virus shedding is minimal. Because a high dose of the vaccine was directly injected intradermally into the snouts of the animals in this study and yet did not cause disease, it is unlikely that vaccination of humans with the rVSV∆G/EBOVGP vector would result in a productive infection with clinical disease in domestic pigs during a spillover event. Moreover, even if this spillover were to occur, the near absence of virus shedding in the rVSV∆G/EBOVGP–infected animals suggests that spillover would not result in maintenance of rVSV∆G/EBOVGP within a pig herd. This study provides data to support the safety of the live-attenuated VSV∆G/EBOVGP vaccine in a relevant livestock species. Should exposure/infection of pigs occur during a vaccination trial in humans, it is highly unlikely that signs of disease would develop in pigs or that the vaccine virus would be disseminated by interspecies or intraspecies transmission.
